# Editorial: Diagnostic, prognostic and predictive factors of response in the era of precision oncology in breast cancer

**DOI:** 10.3389/fonc.2023.1337315

**Published:** 2023-11-23

**Authors:** Diletta Favero, Daniele Generali, Francesco Schettini

**Affiliations:** ^1^ Department of Medical Oncology, IRCCS Ospedale Policlinico San Martino, Genova, Italy; ^2^ Translational Genomics and Targeted Therapies in Solid Tumors Group, August Pi I Sunyer Biomedical Research Institute (IDIBAPS), Barcelona, Spain; ^3^ Breast and Brain Unit, ASST Cremona, Cremona, Italy; ^4^ Department of Medical, Surgical and Health Sciences, University of Trieste, Trieste, Italy; ^5^ Medical Oncology Department, Hospital Clinic of Barcelona, Barcelona, Spain; ^6^ Faculty of Medicine and Health Sciences, University of Barcelona, Barcelona, Spain

**Keywords:** breast cancer, prognosis, prediction, biomarker, precision oncology

Breast cancer (BC) is a multifaceted disease, characterized by distinct molecular subtypes and various biological features with different prognostic and therapeutic implications (1). The prognosis of patients diagnosed with BC has undergone a remarkable transformation in the last twenty years, nonetheless there is still a subset of women who experience a poor response to treatment strategies, and the outcomes for these individuals are frequently unfavorable (2). The examination of the cancer genome and the increasing utilization of next-generation sequencing (NGS) platforms have led to a revolution in our capacity to investigate, diagnose and treat cancers, including breast tumors (3). Specifically, multi-gene tests can now refine the prognosis of early-stage BC patients beyond standard clinicopathological features, supporting also the decision for the treatment escalation or de-escalation (4, 5). It might also be helpful in guiding potential therapeutical approaches in advanced hormone receptor-positive (HR+) disease, at least in the clinical research setting (6–8). Several mutations detectable in tumor tissue or via liquid biopsy can lead to targeted therapeutic treatments, like the novel oral selective estrogen degrader (SERD) elacestrant in *ESR1*-mutant advanced HR+ BC, the selective PI3K inhibitor alpelisib in combination with endocrine treatment in *PIK3CA*-mutant HR+ metastatic BC (MBC) or the small molecules inhibitors larotrectinib and entrectinib in MBC with rare but detectable *NTRAK* fusions (9–12). Nevertheless, although giant steps have been made, much research is still needed to bring a broader personalized molecularly-driven approach to the clinical management of patients affected by breast tumors.

This Research Topic embodies 32 novel studies derived from basic, translational and clinical research in the field of precision medicine in BC, with a particular focus on the discovery of molecular diagnostic and/or prognostic tools as well as predictive biomarkers of response to targeted therapies. Specifically, this Research Topic includes 23 original articles, 5 reviews and 4 case reports, which can be regrouped into four different thematic sections: 1) Predictive tools of response to therapies; 2) Diagnostic and prognostic biomarkers; 3) Diagnostic models based on imaging data; 4) Tailored therapeutic strategies. We will briefly revise the most significant contributions.

## Predictive tools of response to therapies

The first section consists of two original research articles focusing on predictive tools of BC response to oncological treatments.

The importance of achieving a pathological complete response (pCR) and the possibility of adjusting therapies is the Research Topic of the work by Xu et al. in which they identified “Volume change rate” (δV) as a good quantitative efficacy evaluation index to monitor the therapeutic effect of neoadjuvant chemotherapy (NAC) and to predict pCR, to guide the adjustment of individualized NAC regimen. Tashireva et al. conducted a study on a limited cohort of triple-negative breast cancer (TNBC) patients treated with eribulin. They successfully identified immunological predictive markers, including tumor-infiltrating lymphocytes, CD8+, CD4+, FoxP3+, CD20+ lymphocytes, and their PD1 positivity or negativity, associated with treatment response; these findings are consistent with the existing immunomodulant effect of eribulin in addition to its antimitotic effect.

## Diagnostic and prognostic biomarkers

The second section features studies investigating novel clinical or biological markers playing a critical role in the early diagnosis and prognostic assessment of BC, for a more personalized patients care.

The first two articles in this section highlighted the challenge of testing new biomarkers in blood samples.

Zhang et al. focused on the possible role of carnitine compounds in BC development and progression, and in a 1:1 age-matched retrospective case-control study identified increased butyrylcarnitine (C4) levels in whole blood as a risk factor for the disease. The review by Yi et al. is a comprehensive overview regarding small extracellular vesicles (sEVs) and their involvement in BC pathogenesis: the significant role of sEVs in facilitating intracellular communication through the transportation of a variety of biomolecules, with regard to their use as liquid biopsy biomarkers for both the diagnosis and prognosis of BC. Moving on to genomic and transcriptomic techniques, by integrating data of almost 3000 BC patients collected in public database Fan et al. identified a total of 15 hub genes associated with BC long-term survival. Song et al. investigated the role of small nucleolar RNAs (snoRNAs) in tumor development. Their original article, in which they analyzed public database and 77 BC patients’ biopsies, showed a significant up-regulation of SNORA38 and its correlation with tumor size, lymph-node metastasis, and TNM stage. SNORA38 seems to have a relevant carcinogenic role in BC and it was ultimately suggested as a potential prognostic biomarker. Moreover, interestingly, Huang et al. provided a comprehensive analysis of the oncogenic roles of the myelin protein zero-like 3 (MPZL3) pan-cancer gene across different tumors, and its potential role as a prognostic biomarker and therapeutic target for BC.

The importance of the immune system in controlling cancer development, treatment responses and long-term survival of cancer patients by manipulating immune response with several therapeutic immunotherapeutic strategies has already been established or under intensive investigation (13–15); consequently, numerous researchers have directed their efforts toward investigating immune-related genes in the pursuit of developing reliable predictive models for immunotherapy outcomes and patients prognosis. On this Research Topic of Tian et al., Yang et al. and Liu et al. developed prognostic models based on different immune-related genes, which seem to be promising in effectively assist clinicians with medical diagnoses, evaluating patient prognosis and formulating diverse treatment strategies. Moreover, Zhang et al. attempted to assess the immunological and prognostic significance of the V-domain Ig-containing suppressor of T cell activation (VISTA), a crucial immune checkpoint protein, in patients with TNBC. They found that VISTA exhibited a significant correlation with favorable prognosis and increased immune infiltration in TNBC patients. Fan et al. explored the potential connection between molecular subtypes and the preferential distant metastasis sites among BC patients, providing an example of how precision medicine may guide decisions related to surveillance and the development of tailored screening and cancer management strategies for personalized follow-up. Similarly, Cai et al. endeavored to identify high-risk factors for HER2-positive BC patients who would likely develop brain metastasis, a novel approach to support clinicians during the follow-up.

When striving to provide a precision medicine-based treatments, it is imperative to merge it with the clinical and pharmacological anamnesis of the patient. Recent studies have shown a connection between body composition and the prognosis of BC patients. In this context, Liu et al.‘s retrospective study illustrates that visceral obesity is linked to a greater risk of disease recurrence in a Chinese cohort, consistently supporting some already published literature in non-asian populations (16–19). The authors also found sarcopenia was significantly associated with increased recurrence and overall mortality among patients with BC. This underscored and confirmed the significance of body composition assessment as a simple and useful approach to complement the management of BC.

In the last three notable articles of this section, the authors (Zhu et al., Ma et al.) created novel prognostic nomograms by combining various statistically significant variables in order to enhance accuracy in predicting survival of BC patients. Interestingly, Pu et al. focused on predicting the need of chemotherapy in elderly patients solely using clinicopathological data, irrespective of HR, HER2 status, and lymph-node metastasis, without genomic data, with the advantage of being more easily appliable in resource-limited regions or in elderly patients who do not meet indications for genomic tests.

## Diagnostic models based on imaging data

The third section includes three intriguing examples of the role held by imaging techniques in the era of personalized oncology.

Miao et al. evaluated the clinical utility of 68Ga-HER2 affibody PET/CT to non-invasively assess the HER2 expression in BC lesions with uncertain HER2 status, suggesting the potential for this approach to evolve into a personalized “image and treat” strategy for monitoring changes in receptor expression during treatment and optimize therapeutic decisions. Zheng et al. developed and validated a radiomic model based on gray-scale ultrasound and contrast-enhanced ultrasound (GSCEUS) images to effectively differentiate invasive ductal carcinoma from other inflammatory masses, which can help preventing unnecessary biopsies. Sheng et al. showed interesting results from a radiomic machine learning analysis able to integrate the clinical features and the radiomic variables on dynamic contrast-enhanced magnetic resonance imaging (DCE-MRI) to successfully predict distinct molecular subtypes of breast cancer.

## Tailored therapeutic strategies

The last section features papers discussing the potential role of precision medicine in providing personalized treatments tailored to the specific characteristics of both the patient and the tumor.

Over the past years, the role of the immune system in cancer development and progression has gained increasing attention. In their review Gianni et al. identify lymphocytic indexes as new potential prognostic and predictive markers for advanced BC treatment, mainly because of their easiness of detection and applicability in daily clinical practice. Moreover, the authors provided an overview of the possible value of systemic inflammatory cells as therapeutic target or vehicle of treatment. The role of immune checkpoint blockade is addressed by Chen et al., who focus their attention on CD47 as novel attractive target for the treatment of BC. An interesting paper by Xia et al. reported four cases of individualized treatment for advanced BC using the patient-derived tumor-like cell cluster (PTC) model, shedding light on the possible role of this experimental model as an efficient tool for drug resistance screening and for selecting personalized treatments. Another compelling example of precision medicine as a strategy for disease treatment was described by López de Sá et al. The authors reported a case of a metastatic BC patient harboring a *BRAF* V600E mutation that achieved complete response with dabrafenib and trametinib combination. In addition, Wang et al. explored the therapeutic potential of histone deacetylase inhibitors (HDACi) in patients with acquired resistance to endocrine therapy, and Houssiau et al. reported a case of radium-223 treatment in a 59-year-old patient with bone-only metastatic disease. Finally, the role of radiotherapy after BC surgery was explored by Dai et al. and Yang et al. who aimed at developing a nomogram to predict the survival benefit of radiotherapy across various patient groups, with the aim of offering more finely-tailored treatment recommendations.

In summary, this compilation of original articles and reviews provides a valuable insight into prognostic, predictive, diagnostic and therapeutic innovations supporting the implementation of precision medicine in the field of breast cancer treatment. The Editors yearn that the research findings presented in this Research Topic will serve as an inspiration for scientists and clinicians and support the development of clinical trials and breast cancer research, thereby promoting ongoing advancements in personalized care for BC patients in the years to come.

## Author contributions

DF: Writing – original draft. DG: Conceptualization, Supervision, Writing – review & editing. FS: Conceptualization, Supervision, Writing – review & editing.
